# Color Afterimages in Autistic Adults

**DOI:** 10.1007/s10803-016-2786-5

**Published:** 2016-04-28

**Authors:** John Maule, Kirstie Stanworth, Elizabeth Pellicano, Anna Franklin

**Affiliations:** 1 The Sussex Colour Group, School of Psychology, University of Sussex, Pevensey II 5B7, Brighton, BN1 9QH UK; 2 Centre for Research in Autism and Education (CRAE), UCL Institute of Education, University College London, London, UK

**Keywords:** Autism, Afterimages, Adaptation, Color, Top-down knowledge

## Abstract

It has been suggested that attenuated adaptation to visual stimuli in autism is the result of atypical perceptual priors (e.g., Pellicano and Burr in Trends Cogn Sci 16(10):504–510, 2012. doi:10.1016/j.tics.2012.08.009). This study investigated adaptation to color in autistic adults, measuring both strength of afterimage and the influence of top-down knowledge. We found no difference in color afterimage strength between autistic and typical adults. Effects of top-down knowledge on afterimage intensity shown by Lupyan (Acta Psychol 161:117–130, 2015. doi:10.1016/j.actpsy.2015.08.006) were not replicated for either group. This study finds intact color adaptation in autistic adults. This is in contrast to findings of attenuated adaptation to faces and numerosity in autistic children. Future research should investigate the possibility of developmental differences in adaptation and further examine top-down effects on adaptation.

## Introduction

Autism is characterized by difficulties in social communication and behavioral traits including rigid patterns of behavior, preference for sameness, and intense and restricted interests (American Psychiatric Association 2013). Sensory symptoms, including both hyper-reactivity and hypo-reactivity to external stimuli, atypicalities in sensory processing and unusual sensory interests are now included in the most recent revision of the diagnostic criteria for autism (Diagnostic and Statistical Manual of Mental Disorders, Fifth Edition (DSM-5) (American Psychiatric Association 2013). This inclusion implies that they are part of the core of autism, with such atypical sensory experiences potentially also being related to other key features of the condition (Pellicano 2013).

There has been a recent influx of hypotheses attempting to account for these sensory and perceptual differences in particular. Bayesian accounts of perception propose that information from sensory input is combined with prior knowledge or experience to facilitate the interpretation of the sensory information (e.g. Knill and Pouget 2004; Kersten et al. 2004). Pellicano and Burr’s (2012) hypo-priors account posits, within a Bayesian framework, that an under-weighting (relative to typical individuals) of the strength of prior information when interpreting sensory information may underlie autistic sensation and perception. Building on this work, others have proposed accounts of atypical (rather than simply reduced) priors in autism (e.g., Hellendoorn et al. 2015), and predictive coding accounts of autistic perception (Friston et al. 2013; Lawson et al. 2014; Sinha et al. 2014; Van de Cruys et al. 2013).

One sensory process that could be affected by under-weighting of prior information is adaptation (Pellicano and Burr 2012). Adaptation is a fundamental property of neural networks and describes changes in neural activity in response to a persistent stimulus (Kohn 2007). Adaptation is thought to serve a crucial function for sensory systems, tuning neural responses to maximize sensitivity to the range of stimuli present in the immediate environment (e.g. Webster 2011). Adaptation to visual stimuli can result in the experience of aftereffects—distortions in perception which tend to bias perception in the opposite direction to that adapted (e.g. adaptation to a grating tilted to the left, causes a vertical grating to appear tilted slightly to the right (Clifford 2012). Since adaptation afterimages are directly related to the recent sensory input of the beholder, whereby prior sensory experiences are used to calibrate sensory systems and hence bias current perception, adaptation afterimages should therefore be attenuated in autism (Pellicano and Burr 2012).

Several studies have found differences in adaptation aftereffects in autism. Research on high-level face identity aftereffects has shown that they are weaker in autistic compared to typical children (Ewing et al. 2013a; Rhodes et al. 2014), and that the strength of the aftereffect correlates significantly with autistic features, such that those with greater degrees of autistic features have the smallest aftereffects (Pellicano et al. 2007). Subsequent work has shown that adaptation is diminished in autistic children for facial configuration (Ewing et al. 2013b), facial emotion (Rutherford et al. 2012) and eye-gaze direction (Pellicano et al. 2013). There is also evidence for slightly reduced face identity aftereffects in parents (and siblings) of autistic children, suggesting that reduced adaptation may be a feature of the broad endophenotype of autism (Fiorentini et al. 2012). Two studies involving autistic adults, rather than children, have failed to find evidence of reduced adaptation to facial identity and emotion (Cook et al. 2014) and figural contraction/expansion (Walsh et al. 2015), indicating that the differences apparent in childhood may reflect a developmental delay rather than a deficit as such.

More recent work has attempted to determine the pervasiveness of reduced adaptation in autism by examining the nature of the incoming information. In one study, Karaminis et al. (2015) reported that autistic children showed similar levels of adaptation to perceptual causality as typical children. This effect potentially operates at a lower level of the visual hierarchy leading some authors (Turi et al. 2015) to argue that diminished adaptation in autistic people might be confined to high-level stimulus attributes. In support of this claim, Turi et al. showed that autistic children (aged 7–14 years) exhibit less adaptation to numerosity (i.e. number of items present in a part of visual field) than age- and ability-matched typically developing children.

Together, these results are intriguing but further examination of the nature and extent of diminished adaptation in autistic individuals is warranted. Here, we address two key questions—first, do claims for attenuated adaptation in autism generalize beyond face perception to other domains? And, second, do claims for attenuated adaptation in autism generalize from childhood to adulthood? We investigate these issues with regards to adaptation to a low-level visual attribute, color.

Adaptation to color can be demonstrated by the phenomenon of color afterimages—an observer who stares at a colored patch for a few seconds will experience an afterimage of the patch in the opponent color (e.g. afterimage of lime-green following adaptation to magenta) if they look at a uniform white field (Wheatstone 1838). The locus of adaptation which causes color afterimages has been the subject of discussion, and the current evidence suggests that color afterimages are instantiated by adaptation of photoreceptors in the retina and retinal ganglion cells, but may then be subject to further modulation by cortical processes (Zaidi et al. 2012). For example, if an observer is alternately shown a vertical grating on a red background and a horizontal grating on a green background for around 2 min they will subsequently perceive a reddish tinge to a horizontal grating on a white background, and a greenish tinge on a vertical grating on a white background (McCollough 1965). This is known as the McCollough effect, and is most likely mediated by processes in early visual cortex (area V1; Vul and MacLeod 2006), not just at the level of the retina.

The primary aim of the current study was to investigate whether color adaptation is reduced in autistic individuals, as has been shown previously for faces and numerosity. There is already evidence that autistic children differ from their typically developing peers in both color discrimination and memory, at least under some conditions (Cranwell et al. 2015; Franklin et al. 2008; Fujita et al. 2011; Heaton et al. 2008; but see also Koh et al. 2010). There is also anecdotal and case study evidence for enhanced color associations (both phobias and intense interests) in autism (Ludlow et al. 2014) and hyper-sensitivity to certain colors, ambient lighting and colored patterns (Robertson and Simmons 2015). Together these studies suggest that various aspects of color vision and cognition may be different in autistic people. Color adaptation, however, has not been the subject of any studies of autistic perception published to date.

If perception in autism is associated with a generalized reduction in adaptation– as the hypo-priors account predicts—then we would expect to find weaker afterimages for autistic participants compared to typical participants. It is important to note, however, that the initial neural locus of color afterimages is the retina (Zaidi et al. 2012), whereas the prior evidence for attenuated adaptation in autism has all been for higher-level stimuli (faces, numerosity) which are represented in cortical areas (Kanwisher et al. 1997; Piazza and Izard 2009). However, there also is some limited evidence for an association between autism and different patterns of activity in the retina from a study of flash electro-retinogram (ERG) (Ritvo et al. 1988). This study found that rod and cone activity was reduced in 13 out of 27 autistic participants and additional intellectual disabilities, relative to age- and sex-matched typical individuals. Therefore, differences in the basic overall amount of color adaptation in autism may also be predicted on the basis of differential function at the retinal level.

A secondary aim of this study was to investigate whether effects of top-down knowledge on color afterimages is reduced in autism. Lupyan (2015) has claimed that color adaptation is subject to influences from top-down knowledge of a scene or object’s typical color, using a task based on the ‘Spanish Castle Illusion’ (e.g., Sadowski 2006). The ‘Spanish Castle Illusion’ is a color adaptation illusion where exposure to a highly-saturated adapting image, in the complementary colors to the original, causes the observer to see a subsequent greyscale image in the original colors (albeit desaturated). The effect is most striking when the adapting and greyscale image are created from a photograph of a landmark or natural scene. The version which gave the illusion its name used an image of the castle of Manzanares el Real in Spain, which included the castle, with a clear sky forming most of the background, and vegetation in the foreground (see Sadowski 2006). The adapting image is formed by inverting the colors of the base image, flattening the luminance profile and boosting the saturation of the resulting “negative” image. After a short period of fixation (e.g. 20 s), the adapting image is replaced by a greyscale version of the original. The superimposition of the color afterimages with the greyscale contours of the image results in the percept of an image presented in the full, original color (Daw 1962)—an effect which is also observed in illusions of “filling-in” of color afterimages when coupled with luminance contours (e.g. Anstis et al. 2012; Powell et al. 2012; van Lier et al. 2009).

Using a nulling method, whereby participants were asked to adjust the image until it appeared greyscale following adaptation, to measure the strength of the afterimage, Lupyan (2015) found that afterimages were stronger for images of natural scenes (which have typical colors) compared to images of man-made objects (which might be any color). Yet when the same images were turned upside-down in order to disrupt the ease with which the image content could be extracted, the difference between the natural and man-made scenes was abolished. This effect of image orientation on the natural scenes cannot be accounted for by low-level differences in the stimuli used, since it is the same image, rotated through 180 degrees. Lupyan attributed this interaction between image orientation and content as being reflective of top-down influence on the perception of the afterimage—scenes with color-diagnostic content (i.e. typically containing particular colors) are subject to modulation by color knowledge, whereas scenes with non-color-diagnostic content receive no top-down influence. Turning a diagnostic image upside-down disrupts the mechanism responsible for predicting the scene colors, and thus diminishes the effect of color knowledge on the afterimage.

We sought to replicate Lupyan’s (2015) study with samples of autistic and non-autistic adults. We also attempted to improve on Lupyan’s methods by using the two original stimulus images (Experiments 2A–E), in addition to two new images with color-diagnostic and non-color-diagnostic content. Furthermore, we used a perceptually-valid, display-independent stimulus space to measure the strength of afterimages. This paradigm provides an additional exploration of the hypo-priors account of autism, since the effect of top-down knowledge modulating the appearance of afterimages requires the application of priors to predict the appearance of the color-diagnostic scenes. The hypo-priors account would therefore predict that the interaction of image content (i.e. the color-diagnosticity of man-made vs. natural scenes) with image orientation should be weaker, in autistic individuals.

We used the nulling method as described by Lupyan (2015) to investigate two hypotheses related to the hypo-priors account of autism (Pellicano and Burr 2012) and other predictive coding accounts (Friston et al. 2013; Lawson et al. 2014; Sinha et al. 2014; Van de Cruys et al. 2013). This method provides a basic measure of adaptation in addition to potentially allowing the detection of top-down effects on adaptation. The primary hypothesis was that afterimages would be weaker in autistic adults, reflecting attenuated adaptation to visual stimuli. The secondary hypothesis was that autism would be associated with reduced top-down effects on afterimage magnitude.

## Methods

### Participants

Sixteen adults (8 male) diagnosed with an autism spectrum condition took part, recruited through two local autism charities. All adults had received an independent clinical diagnosis of autism (n = 7) or Asperger’s syndrome (n = 9) according to ICD-10 (World Health Organization 1992) or DSM-5 (American Psychiatric Association 2013) criteria. For inclusion in the data analysis participants also had to exceed threshold for autism on the self-report Social Responsiveness Scale-2nd edition (SRS-2) (T-score ≥ 60) (Constantino and Gruber 2012) or the Adult Autism Quotient (AQ; Baron-Cohen et al. 2001) (score ≥ 30, a threshold shown to correspond to a high degree of specificity (i.e. low false-positive rate) and sensitivity (i.e. high hit-rate) (Woodbury-Smith et al. 2005)). Two participants (both male) failed to meet threshold for autism on either questionnaire and were therefore excluded from analysis. Participants were also screened for IQ as an index of their cognitive functioning. One male participant was excluded both due to a low-average full-scale IQ score (of 72) on the Wechsler Abbreviated Scale of Intelligence-Second Edition (WASI-II; Wechsler and Psychological Corporation 2011) and for failure to follow the task instructions, another male participant gave responses consistent with a color vision deficiency on the Ishihara test (Ishihara 1973) and was excluded, as was another female participant who did not follow the task instructions. A final sample of 11 adults (4 male) formed the autism group. The gender ratio in this sample is unusual, given the male preponderance in diagnosed cases of autism.

Sixteen typical adults were recruited from community contacts. Data from two participants were removed as they scored over 60 on the SRS-2. Three further participants were excluded for matching purposes, yielding a final sample of 11 typical adults (4 male). The final groups of typical and autistic participants were well-matched at the group level in terms of gender proportion, mean age and mean IQ, but, as expected, differed significantly on mean SRS-2 and mean AQ score (see Table 1).

**Table 1:** Descriptive statistics and group comparisons for age, IQ, AQ and SRS-2 for autistic and typical adults

Measure	Group				Group difference	
	Autistic adults		Typical adults		*t* test	Cohen’s d^a^
	Mean (SD)	Range	Mean (SD)	Range		
Age in years	24.8 (4.9)	19–34	23.9 (4.8)	19–33	*t*(20) = 0.44, *p* = 0.668	0.20
Full-scale IQ^b^	108.6 (12.6)	82–133	109.6 (8.1)	95–121	*t*(20) = 0.22, *p* = 0.827	0.10
VCI^c^	104.2 (12.3)	84–126	109.0 (12.7)	91–132	*t*(20) = 0.89, *p* = 0.385	0.40
PRI^d^	111.7 (13.8)	91–132	107.9 (8.3)	92–121	*t*(20) = 0.79, *p* = 0.441	0.35
AQ^e^	37.7 (5.9)	29–49	15.0 (5.4)	7–23	*t*(20) = 9.45, *p* < 0.001	4.23
SRS-2^f^	78.0 (6.9)	68–90	49.9 (9.4)	26–60	*t*(20) = 8.00, *p* < 0.001	3.58

^a^Cohen’s d computed from *t* test results, as recommended by Kover and Atwood (2013) for matched-group designs

^b^IQ = Intelligence Quotient, as measured by the four sub-tests of Wechsler Abbreviated Scale of Intelligence-II (WASI-II; Wechsler and Psychological Corporation 2011)

^c^VCI = Verbal Comprehension Index, verbal sub-scale of WASI-II

^d^PRI = Perceptual Reasoning Index, non-verbal sub-scale of WASI-II

^e^AQ = Adult Autism Quotient (Baron-Cohen et al. 2001)

^f^SRS-2 = Adult Social Responsiveness Scale-2nd edition (Constantino and Gruber 2012). This table reports standardized (age-adjusted) scores on the full-scale IQ, PRI, VCI and SRS-2; the statistical inferences about the differences between the groups are the same for raw scores

All 22 participants reported normal or corrected-to-normal visual acuity and were assessed as having normal color vision using Ishihara plates (Ishihara 1973) and the Lanthony tritan test (Lanthony 1998). Participants were paid standard rates of £7.50 per hour. All procedures performed in studies involving human participants were in accordance with the ethical standards of the institutional and/or national research committee and with the 1964 Helsinki declaration and its later amendments or comparable ethical standards. The research protocol was approved by the University of Sussex Sciences and Technology Cross-School Research Ethics Committee (approval reference ER/JJM29/3) and all participants gave written informed consent prior to participation in this study.

### Stimuli

**Fig. 1:**
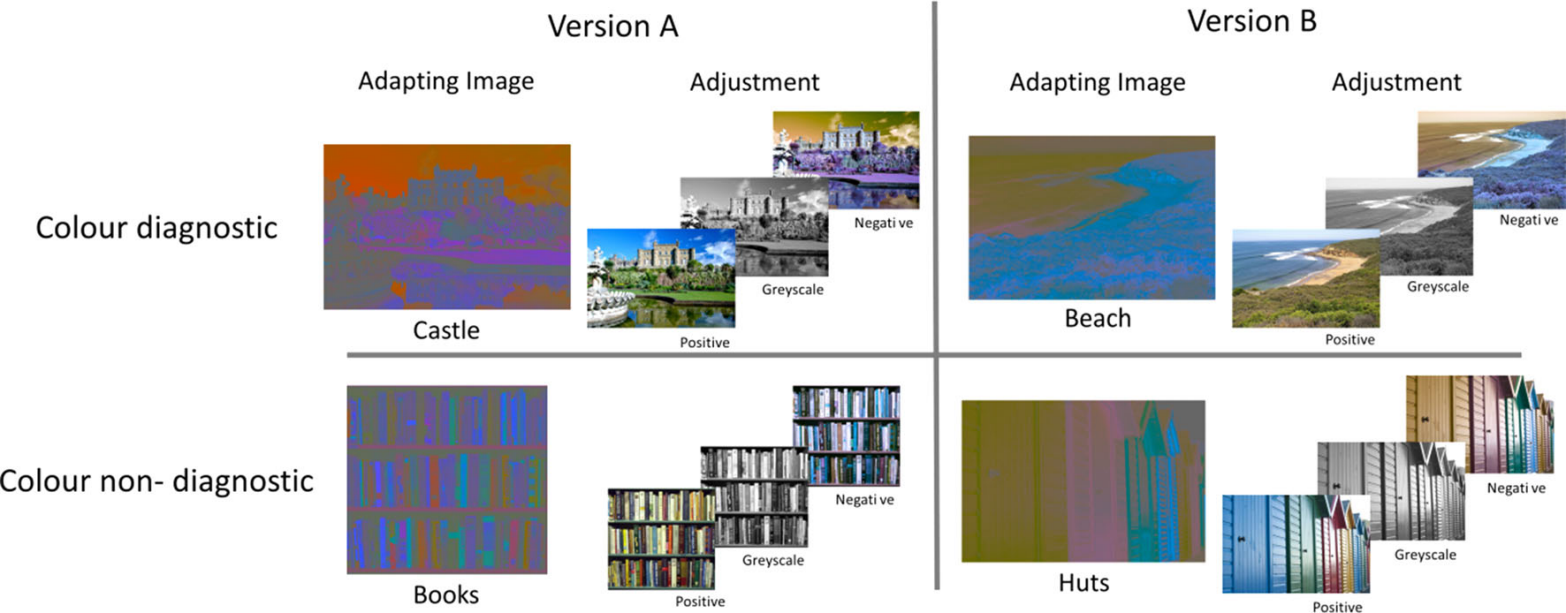
Images used in the experiment. At the adjustment phase a continuum of 51 images of gradually changing color intensity between the *positive*, *greyscale* and *negative images* was available for selection. The dimensions of the images varied slightly due to the proportions of the source images

Four photographs were used as the basis for all stimuli presented in the experiment. Each photograph depicted one of the following four scenes: books on shelves (“books”); Culzean Castle in Scotland (“castle”); Bells beach, Australia (“beach”); and a row of painted, wooden beach huts in Whitby, England (“huts”). The books (color non-diagnostic) and castle (color diagnostic) images were identical to those used by Lupyan while the huts and beach photographs were selected as good examples of images which are color-diagnostic (beach) or color non-diagnostic (huts)^1^ (see Oliva and Schyns 2000) (Fig. 1).

**Fig. 2:**
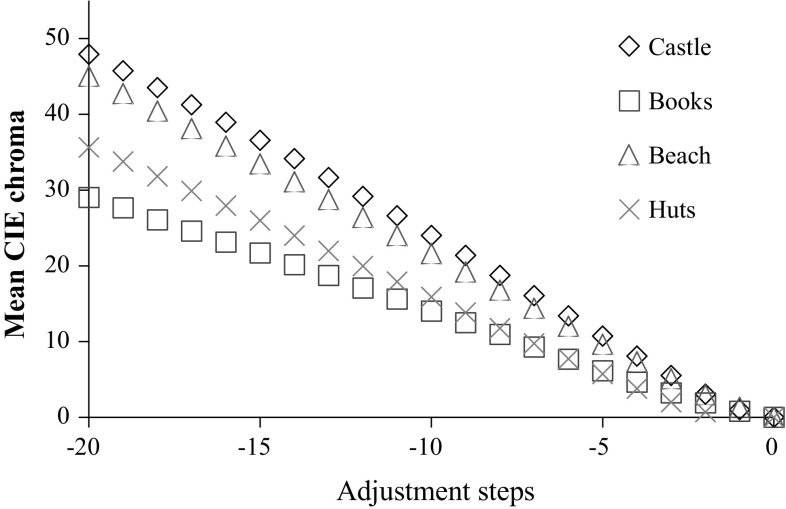
Mean difference in whole-image chroma from the greyscale image (0 on the x-axis), for the adjustment steps for each stimulus image. Although the adjustment scale included 51 steps from −25 to +25, the actual range of settings chosen by observers was −21 to +3, and 95 % of trials the setting chosen was in the range −20 to 0

The adapting stimuli were generated following the Adobe Photoshop actions described in John Sadowski’s online demonstration of the Spanish Castle illusion (Sadowski 2006). The procedure involves the flattening of the luminance profile of the image, inverting the colors in HSL space and enhancing the color intensity of the resulting image. These adapting images are not equated in color saturation, so the image orientation manipulation is used to disentangle the effect of the image content from low-level differences in the adapting stimuli. Since the rotation through 180 degrees does not change the low-level properties of the image, if aftereffects are solely determined by the intensity of the colors in the adapting image, one would expect orientation to have no effect on the magnitude of the aftereffect. In contrast, if the afterimage magnitude is affected by top-down knowledge this may be disrupted by rotation (Lupyan 2015). Therefore, the orientation manipulation provides a control for differences in the low-level properties of the adapting images, and allows for the detection of purported top-down effects on afterimage strength. Inverted and greyscale versions of each original image were also generated using the hue rotation and desaturate functions of Photoshop in HSL-mode. For each of the four basic images, a smooth continuum consisting of 51 images between the original, “positive” image, through the greyscale image and to the inverted “negative” image were created through linear transformation of the RGB values for each pixel.^2^ This continuum was the basis for the adjustment phase of each trial. It is important to note that this stimulus space is arbitrary and does not necessarily represent homogeneous or equal steps in terms of perceptual difference. Comparisons across images (e.g., comparing books to castle) in terms of these raw units should therefore be avoided, particularly as there is some variation in the perceptual size of steps across images. Figure 2 illustrates this variation—the relationship between the adjustment scale and mean saturation of the image is linear, but there is a small amount of variation in the perceptual difference that each step represents across images (see the differences in slope). The mean (SD) step size in CIE chroma units for each image was as follows: castle—2.40 (0.36); books—1.45 (0.19); beach—2.25 (0.27); huts—1.81 (0.45). To address the issue of non-comparable units on the adjustment scale, after the experiment, the continuum images were subsequently re-coded according to their difference from greyscale using CIE chroma, a perceptual color space—this is further explained in the results section. Further details of the colormetric properties of the stimulus images are given in Table 2. Throughout the experiment, a background grey with chromaticity equivalent to D65 (CIE (1931) xyY = 0.313, 0.329, 20) was used.

**Table 2:** Colorimetric properties of the original (positive), inverted (negative), and adapting images used in the study

	Image	Mean CIE (1931) Y (range)	Mean CIE chroma (range)
Positive image	Castle	17.43 (0.00–62.19)	66.71 (0.00–191.38)
	Books	15.76 (0.00–61.60)	37.69 (0.00–244.01)
	Beach	19.32 (0.00–62.19)	52.05 (0.00–116.55)
	Huts	22.31 (0.02–62.19)	59.39 (0.15–135.64)
Negative image	Castle	17.62 (0.00–62.19)	60.19 (0.00–172.99)
	Books	16.05 (0.00–61.49)	41.72 (0.00–173.72)
	Beach	19.47 (0.00–62.19)	58.73 (0.00–156.65)
	Huts	22.27 (0.06–62.19)	45.82 (0.13–164.44)
Adapting image	Castle	9.46 (7.91–14.59)	81.44 (0.73–244.02)
	Books	9.40 (8.21–16.46)	61.37 (0.80–217.62)
	Beach	9.78 (7.60–18.72)	73.44 (0.73–152.59)
	Huts	9.39 (7.18–17.84)	50.58 (0.73–148.95)

All values are a summary of all pixels in each image. CIE—International Commission on Illumination; Y—luminance in cd/m^3^. In most images the range of pixels includes some white and some black pixels, therefore the range of Y and chroma is fixed at the limits of the monitor used. For the adapting images, however, part of the process to create them involved a flattening of the luminance profile, meaning that these images have a more restricted range of luminance

These images (in all colored and achromatic forms) were always presented centrally on the monitor with a fixation cross in the center. All images subtended 25° of visual angle horizontally and 17° (24° for the books image) of visual angle vertically, and were presented on a grey background. The background subtended 39° of visual angle horizontally and 29° of visual angle vertically.

### Apparatus

The adaptation tasks were completed on a 22-inch Mitsubishi DiamondPlus 2070SB Diamondtron CRT monitor, with a resolution of 1600 × 1200 pixels, 24-bit color resolution, and a refresh rate of 100 Hz. Participants made their responses using the appropriate keyboard press. A ColorCal colorimeter (Cambridge Research Systems) was used to measure the monitor and calibrate the primary values (RGB) for the background color regularly during the data collection period. The tasks took place in a blacked-out room, with the monitor as the only source of light. A cardboard viewing tunnel lined with black felt was used to eliminate effect of peripheral objects and colors and a chin rest was used to constrain viewing distance at 57 cm, ensuring consistency of the perceived size of the images and overlap of the after-images with adjustment image.

### Design

The experiment involved two versions of an adaptation task, both identical in procedure, length and task, but with different images in each. Version A included the books and castle images and version B the huts and beach images. Each version had two images—one color diagnostic (castle/beach) and one color non-diagnostic (books/huts). In each version one image would be presented upright (hereafter referred to as “up”), and the other upside-down (hereafter “down”). This diagnosticity-orientation pairing was reversed in the second version (e.g. version A—castle up, books down; version B—huts down, beach up). Thus, each participant would adapt to color-diagnostic scenes in both orientations and color non-diagnostic scenes in both orientations. The order in which the participant completed the versions (A/B) was also counterbalanced across participants.

For the purpose of analysis the trials were classified according to the class of the image (i.e. books and huts are “non-diagnostic” and castle and beach are “diagnostic”) and orientation (upright or upside down), such that, across both versions of the adaptation nulling experiment, each participant provided settings for four conditions—diagnostic upright, diagnostic upside-down, non-diagnostic upright and non-diagnostic upside down. The specific image used in each condition varied between participants (e.g. for some the diagnostic upright image was the castle but for others it was the beach) but was counterbalanced across groups.

### Procedure

Participants were seen individually at the university either for a single session lasting approximately 2 h or for two shorter sessions. Within the session, the participant completed one experimental task followed by the two questionnaires tapping autism symptomatology (the AQ and SRS-2) and the WASI-II, followed by the second experimental task.

Prior to beginning the task proper, participants were briefed with instruction sheets explaining the trial procedure and the participant’s task. These instructions encouraged the participants to try to ‘make their adjustments as quickly and accurately’ as they could and emphasized the importance of maintaining a steady gaze, fixed on the central cross.

To begin, participants completed eight ‘norming’ trials, in which there was no adaptation phase, only adjustment to greyscale (trials started at a random point on the positive–negative color continuum). Four norming trials for each stimulus image were completed, with the image only displayed in the orientation it would be presented in for the adaptation trials. Norming was carried out for each image that would be subsequently presented in the adaptation trials. This was ostensibly for practice, allowing participants to become accustomed to the adjustment procedure and range of stimuli, but also allowed recording of the pre-adaptation settings for each image and observer (following Lupyan 2015).

Next, participants were presented with version A or B of the experimental task. Each experimental trial required participants to stare at a fixation cross in the center of an adapting (color) image for 20 s, which was immediately replaced by a greyscale version of the same image in the same orientation. A nulling procedure was used to measure the strength of the afterimage in terms of the shift in perception of this greyscale image towards the positive (natural) colors of the photograph. The participant was instructed to adjust the image until it appeared subjectively greyscale. This was done using the up and down arrow keys on the keyboard, which were assigned at random on each trial to either adjust the image towards the positive-color image or the negative-color image along the adjustment continuum. When the participant felt the image was greyscale they pressed the space bar. Participants were given 30 s between each trial to allow for the dissipation of the color aftereffects.

Each version consisted of eight adaptation trials (four for each of two images, of which one image would be upright and one upside down) presented in a pseudo-random order, with the constraint that trials presenting the same image could only appear a maximum of two times in succession. After having completed one version of the task (version A or version B), participants filled in the AQ and SRS-2 self-report questionnaires, then completed the second version. The order of versions was counter-balanced across participants.

## Results

Settings were coded initially by an index number that referred to the selected image from the adjustment continuum. However, in order to compare across images it was important to approximate the perceptual equivalence of these indices. The CIE L*u*v* color space is a perceptually-uniform description of the colors visible to adult human observers. It includes parameters that approximate to saturation or chromatic intensity called “chroma”, as well as hue and lightness. Stronger afterimages require more intense chromatic input to null them. The chroma of each image selected in the experiment to null the aftereffect was subtracted from the chroma of the presented greyscale image that was in the center of the adjustment continuum. This was calculated first for each pixel and then this difference was averaged across the whole image to provide the mean chroma difference from grey for each image. Trials where the selected image was in the opposite direction to that expected given the adaptation image (i.e. towards the positive image) were given a negative chroma score. This allowed us to compare different images using a perceptually meaningful measure. Higher chroma difference scores indicate that more intensely-colored images were selected to null the afterimage, indicating that the afterimage was stronger in that trial or condition.

The pre-adaptation settings were first analyzed for group differences (to make sure both groups could set to greyscale) and to confirm that pre-adaptation subjective greyscale points had mean CIE chroma no different from zero. A 2 (image type: diagnostic/non-diagnostic) × 2 (orientation: upright/upside-down) × 2 (group: autism/typical) mixed ANOVA was run on the pre-adaptation chroma settings. There was a marginal, but non-significant main effect of image type (*F*(1,20) = 4.13, *p* = 0.056, *r* = 0.41), and a marginal, but non-significant interaction of orientation with image type (*F*(1,20) = 4.35, *p* = 0.050, *r* = 0.42). One-sample *t* tests indicated that the mean chroma of images selected as appearing subjectively greyscale in the pre-adaptation settings did not deviate significantly from zero in any of the four conditions (natural up, man-made up, natural down, man-made down) (largest *t* = 1.75, smallest *p* = 0.095). There were no other significant main effects or interactions. There was no effect of autism on the achromatic settings (*F*(1,20) = 1.40, *p* = 0.250, *r* = 0.26). Therefore both groups were equally able to set the images to greyscale, and there was no difference in the settings across image type and orientation.

Following Lupyan’s finding, we predicted that afterimages would be stronger (i.e. require more nulling) for diagnostic than non-diagnostic scenes, but only in the upright condition. We also predicted that that the effect of color diagnosticity on afterimage strength would be absent or attenuated in autistic adults compared to typical adults. Finally, if adaptation is generally reduced in autism, afterimages should be generally weaker across all conditions.

**Fig. 3:**
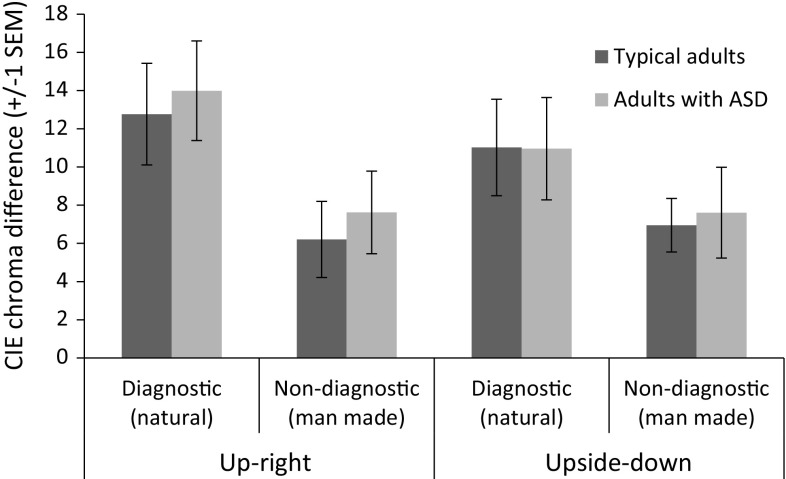
Mean whole-image chroma settings for nulling afterimages following adaptation (in CIE chroma units). The diagnostic images were of a castle and beach, while the non-diagnostic were of books and painted huts. *Error bars* show ±1 SEM

A preliminary analysis confirmed that the settings for each diagnosticity and orientation were consistent across the different images (largest *t* = 0.97, smallest *p* = 0.343), allowing us to collapse across diagnostic (castle and beach) and non-diagnostic (books and huts). Mean chroma differences were submitted to a 2 (image type: diagnostic/non-diagnostic) × 2 (orientation: upright/upside-down) × 2 (group: autism/typical) mixed ANOVA. The only significant effect was a main effect of image type (*F*(1,20) = 29.25, *p* < 0.001, *r* = 0.77). A follow-up *t* test on diagnostic and non-diagnostic images (collapsed across orientation and group) found that nulling settings were significantly more chromatic for diagnostic (M = 12.18, SEM = 1.67) than non-diagnostic images (M = 7.09, SEM = 1.26; *t*(21) = 5.53, *p* < 0.001, *r* = 0.77). Unexpectedly, there was neither a main effect of orientation (*F*(1,20) = 1.95, *p* = 0.178, *r* = 0.30) nor a main effect of group (*F*(1,20) = 0.08, *p* = 0.781, *r* = 0.06). There were also no significant interactions of orientation with image type (*F*(1,20) = 1.53, *p* = 0.231, *r* = 0.27), orientation with group (*F*(1,20) = 0.50, *p* = 0.489, *r* = 0.16), image type with group (*F*(1,20) = 0.06, *p* = 0.811, *r* = 0.05), and no three-way interaction (*F*(1,20) = 0.02, *p* = 0.905, *r* = 0.03) (Fig. 3).^3^

Bayesian statistics allow inferences to be made about the null, in addition to the alternative, hypothesis. A Bayesian approach was used to assess the strength of the evidence [i.e. the probability of the null hypothesis being true, given the data, *p*(H_0_|D); or the probability of the alternative hypothesis being true, given the data, *p*(H_0_|D)] for the crucial three main effects (orientation, image type and group) and the image type × orientation interaction. This was based on calculating the Bayesian information criterion (BIC) following the methods and materials provided by Masson (2011). The effect of image type returned a very high BIC value (ΔBIC = −16.74) indicating very strong support for the alternative hypothesis (*p*(H_1_|D) > 0.999). Support for the null over the alternative hypothesis was observed for the main effect of orientation (ΔBIC = 1.05, *p*(H_0_|D) = 0.628) and for the interaction between orientation and image type (ΔBIC = 2.09, *p*(H_0_|D) = 0.676). Probabilities below 0.75 indicate ‘weak’ support for the null hypothesis in these cases (Raftery 1999), whereas the main effect of group offers stronger support, with a Bayes factor above 3 (Dienes 2011) and ‘positive’ support for the null (Raftery 1999) (ΔBIC = 3.00, *p*(H_0_|D) = 0.818).

One important factor which affects the magnitude of aftereffects is time since the offset of the adapting stimulus. Afterimages fade with time, so the magnitude recorded in this study is directly related to the time spent adjusting each image. Accordingly, participant mean reaction times (RTs) showed a strong negative correlation with mean afterimage magnitude (*r*(20) = −0.53, *p* = 0.01), indicating that those who took longer to make their adjustments tended to report weaker afterimages. Participants’ RTs were submitted to a 2 (image type: diagnostic/non-diagnostic) × 2 (orientation: upright/upside-down) × 2 (group: autism/typical) mixed ANOVA (as was carried out for the aftereffect magnitude data). This analysis revealed no significant main effects or interactions (largest *F* = 2.88, smallest *p* = 0.105—image type), indicating that RTs were consistent across conditions and groups (overall mean RT = 6.34 s).

## Discussion

### Intact Adaptation to Color in Autistic Adults

The primary aim of the present study was to investigate whether color adaptation is reduced in autistic adults. Evidence for attenuated adaptation to faces (Ewing et al. 2013a, b; Fiorentini et al. 2012; Pellicano et al. 2013; Rhodes et al. 2014) and number (Turi et al. 2015) in autistic children may reflect a generalized reduction in adaptation in autism, as the hypo-priors account of autism (Pellicano 2013; Pellicano and Burr 2012) would predict. Contrary to predictions, there were no significant group differences observed in the strength of color afterimages following adaptation to whole-scene images in our sample of autistic adults, relative to a group of typical adults.

There are a number of implications of this finding, relating to the understanding of perceptual atypicalities in autism, and the interpretation of this study’s results. First, the results of the Bayesian analysis shows that the present study provides positive support for the null hypothesis (Dienes 2011; Raftery 1999) in relation to the effect of autism on basic adaptation magnitude. Similarly, the effect size associated with the group factor is very small, indicating a high degree of overlap in the distributions of adaptation strength across the autistic and typical groups. Therefore, we are confident that these findings are reliable.

Second, the basic phenomenon of color afterimages is attributable to adaptation very early on in the visual stream, at the level of photoreceptors and ganglion cells in the retina (Zaidi et al. 2012), whereas the effects of luminance contours which help to make the Spanish Castle effect so vivid reflect the contribution of areas of visual cortex modifying the afterimage signal received from the retina to create the perception of filling-in (Anstis et al. 2012; van Lier et al. 2009; Vergeer et al. 2015). In contrast, previous demonstrations of attenuated adaptation in autism have been for faces and number, which are processed at a higher level, in regions of the temporal (i.e., fusiform face area—Kanwisher et al. 1997) and parietal cortex (see Piazza and Izard 2009, for a review), respectively. In the current study, no general reduction in afterimage strength was observed in the autistic relative to the typical group. This may suggest that only higher-level adaptation is attenuated in autism, where priors and top-down influence may have more impact on perception, whilst low-level visual adaptation mechanisms in autistic adults are intact.

Third, the present study has tested autistic adults, while the majority of previous work demonstrating attenuated adaptation has been in children. For example, whilst children on the autism spectrum may have atypical adaptation aftereffects for facial identity and expression (Ewing et al. 2013b; Rhodes et al. 2014), afterimages in autistic adults are no different in strength to those of typical adults (Cook et al. 2014; Walsh et al. 2015). These latter results are, however, at odds with the finding that the parents of autistic children also exhibit reduced face aftereffects (Fiorentini et al. 2012), suggesting that attenuated adaptation of certain stimuli may also be reduced in genetically-related family members, and is manifest even in typical adults. It is important to note that while Fiorentini et al. found that the effect generalizes to adults who are relatives of autistic children, the effect is small. The evidence for reduced adaptation to numerosity (Turi et al. 2015) is also restricted to autistic children, with no similar study published on autistic adults.

There are relatively few studies which have examined adaptation in autistic adults. It remains possible that color adaptation is atypical in autistic children, even if not attenuated in adulthood. Children have a more limited experience of the world than adults and may not yet have developed compensatory strategies for any perceptual atypicality they experience. Indeed, some studies of typically developing children have found differences between children and adults in afterimages of facial identity and attractiveness, under some conditions (e.g., Hills et al. 2010; Jeffery et al. 2010, 2011). These studies suggest that children have more flexible norm-based coding of faces, due to their more limited experience of the world (see Jeffery and Rhodes 2011 for a review). Other studies have found afterimage magnitude is comparable between adults and children for face aftereffects (Anzures et al. 2009; Nishimura et al. 2008, 2011; Pimperton et al. 2009). The effect of age on color afterimages has not been widely researched. There is a suggestion that afterimages persist for longer in older people in the typical population (Kline and Nestor 1977) but whether the intensity of color afterimages changes with age is unknown. Further studies investigating adaptation in both the typical and autistic populations at different stages in development for a range of perceptual domains are needed to fully examine the question of whether age affects the intensity of adaptation afterimages.

Since we found no difference in adaptation, we fail to support the suggestion of suppressed retinal activity in the retina of autistic individuals, at least in adults (Ritvo et al. 1988). Likewise, there is no evidence that the purported differences in chromatic discrimination in autistic individuals (Cranwell et al. 2015; Franklin et al. 2008; Fujita et al. 2011; Heaton et al. 2008) has any effects on the responses made on this task. While it is the case that poorer discrimination may lead to more variability in achromatic settings, it is unlikely that this would represent a systematic shift of responses. Furthermore, differences in chromatic discrimination do not necessarily imply differences in adaptation. Finally, in adults, differences in chromatic discrimination may be associated with non-verbal ability [at least when measured using the Farnsworth-Munsell 100-Hue Test (FM100) (Cranwell et al. 2015)], whereas our groups are matched for Perceptual Reasoning Index (PRI), the non-verbal sub-scale of the WASI-II (see Table 1). The present study, it should be noted, cannot contribute to the question of chromatic discrimination in autism since the measure used is likely to be relatively insensitive and there are competing task demands related to nulling the adaptation afterimage.

The data from the present study show typical color adaptation in a group of autistic adults, contrasting to evidence for attenuated adaptation in other domains associated with autism in children. This may be due to the lower-level sensory nature of color adaptation compared to those domains in which adaptation has been shown to be attenuated in autism, or due to developmental changes in adaptation between childhood and adulthood. Further research should seek to clarify the role of top-down knowledge on color adaptation in both children and adults with and without autism. Another potentially fruitful approach may be to measure multiple forms of adaptation (e.g. color, motion, faces etc.), with the same task and procedure for each stimulus domain, within individuals in order to compare adaptation across domains and levels of representation.

### Top-Down Effects on Afterimages

Our secondary hypothesis related to a possible effect of scene content on color adaptation. Lupyan (2015) proposed that there are top-down effects of knowledge of a scene on the strength of adaptation and that natural scenes therefore have stronger afterimages than man-made scenes, which are not color diagnostic. While we observed that natural scenes elicited stronger afterimages than man-made scenes, and this effect was similarly strong across groups, we nevertheless failed to replicate Lupyan’s finding of an interaction of this effect with the orientation of the image. This interaction between orientation and image content is crucial to claims of top-down knowledge affecting afterimage magnitude, since effects of image content alone may be attributed to low-level properties of the different adapting images (greater luminance or chromatic intensity). Afterimages in our study were of equivalent strength regardless of whether the adapting image was upright or upside down. This lack of an interaction suggests that the difference in adaptation for type of scene may well be due to low-level differences in the images (e.g., chroma) rather than top-down effects of color diagnostic scene content. The lack of a verified top-down effect on color adaptation in our study means that, unfortunately, we are unable to determine whether effects of knowledge on adaptation are reduced in autistic adults, since we do not find such effects in the group of typical adults.

The failure to replicate Lupyan’s (2015) top-down effect may be due to a number of factors. The method and the stimuli were very similar to Lupyan’s and the samples were of a similar age (early adulthood). Nevertheless there were some methodological differences between studies, which may help explain the discrepancy between the findings. First, the after-effect was measured in different units across studies. Specifically, we quantified the after-effect using the perceptually meaningful measure of chroma rather than units in computational HSL space. However, when we analyzed our results in terms of HSL steps, we still found no effect of orientation and no orientation-diagnosticity interaction.

Second, Lupyan’s diagnosticity-orientation interaction was derived from comparing participants from different experiments, and therefore, *different* participants, while in our study all participants completed all conditions. Seeing both upright and inverted images within one experiment, even if the images are different, could weaken the extent to which the observer views the upright images as a meaningful scene, and/or facilitate the interpretation of upside-down images leading to some top-down effects. Either of these possibilities would lead to a reduction in the strength of the interaction between image content and orientation. If this is true, top-down effects of knowledge may have been weaker in our study than Lupyan’s because of our within-subjects rather than between-groups manipulation of orientation. Notwithstanding, we feel that the within-subjects design of our study is preferable, since it avoids the possibility of cohort effects, where one group happens to have stronger afterimages than the other, and has greater power by virtue of being based on within-participant factors.

Third, the average time taken for participants to make the nulling adjustment is another potential factor. In the present study, we found no relationship between mean response times and the mean measured afterimage magnitudes (i.e. mean response times to all conditions are similar, see results). Lupyan (2015) reported an average adjustment time of just under 10 s, while our participants appear to have made their adjustments more rapidly, the average adjustment taking around 6 s. With time, the strength of the afterimage fades (e.g., Kline and Nestor 1977), it is therefore likely that Lupyan’s (2015) participants were making their achromatic settings for relatively weaker afterimages, compared to the participants in the present study. It is possible that these effects are somewhat sensitive to the time-course of adaptation—perhaps being more likely to be evident when the observer is adjusting to null a less intense afterimage (later) than when the afterimage is stronger (earlier). A weaker afterimage may cause the observer to be less certain of the bottom-up sensory input, and thus more likely to rely on top-down information in making their judgment.

Further research into these factors (within-participant orientation manipulations and response time) may help to clarify whether these aspects of the task design are crucial to identifying top-down effects. However, if it is to generate further interesting and testable research questions about top-down effects on visual perception, the effect should be generalizable beyond the specific parameters of the original study, to other stimuli, paradigms and designs. Our study adds a somewhat cautious view of the generalizability of Lupyan’s effect.

Finally, it is important to note that the Bayesian analysis confirms that the data provide only ‘weak’ (Dienes 2011; Raftery 1999) support for the null hypothesis relating to main effects of orientation and image type and the interaction between these factors. Considering Fig. 3, the aggregate data do appear to tend towards the expected direction of the effect—afterimage intensity is reduced by turning the image upside down to a greater extent for the diagnostic scenes than for the non-diagnostic. However, this trend does not appear at the individual level, with only a few participants’ individual data revealing this expected pattern. It appears from our study that the effect of top-down knowledge of afterimage strength, if present, is relatively subtle (see also Hansen et al. 2006; Olkkonen et al. 2008). The present task may not be the most sensitive test of these effects, and an alternative task which evokes stronger and more reliable effects should be sought to better test predictions relating to top-down influences on perception.

Other studies have shown the effect of prior knowledge or expectation on visual perception. An auditory label which is congruent with an image otherwise suppressed from visual awareness by inter-ocular rivalry can improve detection speed and sensitivity, and is specific to the object (e.g. hearing “pumpkin” speeds detection of pumpkins but not chairs) (Lupyan and Ward 2013). Similarly, when asked to adjust a familiar and prototypically-colored object to grayscale, observers appear to overcompensate for the influence of memory colors. For example, observers’ grayscale settings for a banana tend to be slightly biased in the blue direction, apparently to counteract the influence of the yellow memory color (Hansen et al. 2006; Olkkonen et al. 2008), although some of these top-down effects are notably small. As such, the brain appears to use prior experience of familiar objects with distinct colors to predictively code their appearance (Bannert and Bartels 2013). If such effects reflect top-down modulation of the cortical representation of those objects through the application of priors to current percepts, then we should find individual differences in the strength of these effects depending on autism symptomatology, age and experience with the objects in question. In order to fully explore this possibility, however, one must first find a perceptual task for which a reliable effect of top-down knowledge can be demonstrated in a typical group.

## Conclusion

The main contribution of this paper is to show that there were no differences between autistic adults and typical adults in the overall strength of adaptation afterimage in this task. This study therefore provides evidence for typical color adaptation in autism. Unfortunately, we were unable to address fully the secondary hypothesis regarding the role of top-down knowledge on perception in autism, due to the failure to replicate the top-down effects on afterimage strength in this task reported by Lupyan (2015).

The present study is the first to demonstrate typical color adaptation in a sample of autistic adults, and is contrary to the evidence for attenuated adaptation in other domains of visual perception (Ewing et al. 2013a, b; Fiorentini et al. 2012; Pellicano et al. 2013; Rhodes et al. 2014; Turi et al. 2015). We suggest that this may be due to the early level at which color adaptation occurs and/or developmental changes in adaptation.

## Footnotes

1 To confirm that the stimuli were appropriately classified as color-diagnostic, an informal survey was carried out in which naive typical observers were shown original and inverse-colour versions of stimuli from the experiment, one at a time, and asked “is this image presented in original or altered colors?” Observers did not see both the original and inverted versions of the same base image. The results from 8 observers (two male; mean age 32 years) confirmed that the original castle and beach scenes were always correctly categorised as unaltered (and the inverse versions always categorised as altered), indicating the diagnostic nature of the colour of those scenes. The books and huts images were more ambiguous.

2 All of the stimulus images, including original, negative, adapting and all 51 steps of the adjustment continuum are available from the authors, on request.

3 We also performed an additional analysis with task order entered as a factor. We found no main effect of task order or interaction with other factors (all *p*s > 0.320).
